# Adaptive Evolution of the PFK Gene Family in Chinese Longsnout Catfish, *Leiocassis longirostris*

**DOI:** 10.3390/biology15171481

**Published:** 2026-09-01

**Authors:** Junwei Zhang, Qisheng Lu, Haokun Liu, Zhimin Zhang, Junyan Jin, Shouqi Xie, Dong Han

**Affiliations:** 1 Fisheries College, Ocean University of China, Qingdao 266071, China; zhangjunwei@stu.ouc.edu.cn; 2 State Key Laboratory of Breeding Biotechnology and Sustainable Aquaculture, Hubei Hongshan Laboratory, Institute of Hydrobiology, Chinese Academy of Sciences, Wuhan 430072, China; luqisheng@ihb.ac.cn (Q.L.); liuhaokun@ihb.ac.cn (H.L.); zhangzm@ihb.ac.cn (Z.Z.); jinjunyan@ihb.ac.cn (J.J.); sqxie@ihb.ac.cn (S.X.); 3 College of Advanced Agricultural Sciences, University of Chinese Academy of Sciences, Beijing 100049, China

**Keywords:** *Leiocassis longirostris*, comparative genomics, glycolysis, gluconeogenesis, phosphofructokinase (PFK)

## Abstract

The Chinese longsnout catfish is a typical carnivorous fish with a relatively weak ability to utilize glucose. However, the genomic basis for its glucose metabolic adaptation remains unclear. In this study, we used comparative genomics methods to systematically analyze the evolutionary characteristics of glucose metabolism-related genes in the Chinese longsnout catfish, focusing on gene family evolution, patterns of expansion and contraction, and selective pressures. The results indicate that glucose metabolism-related genes have undergone significant reshaping during evolution. Genes involved in glucose digestion, absorption, and insulin signaling pathways demonstrate a tendency toward contraction, while those associated with protein and lipid metabolism exhibit expansion. This pattern is consistent with the species’ long-term adaptation to a high-protein, high-fat diet. Comparative analysis further revealed that, compared to fish with different dietary habits, certain key genes involved in glycolysis in the Chinese longsnout catfish exhibit a reduction in copy number. Molecular evolutionary analysis showed that key genes involved in glycolysis and gluconeogenesis (including hexokinase 2 (*hk2*), phosphofructokinase, muscle/platelet (*pfkm*/*p*)) exhibit signs of accelerated evolution or positive selection. Notably, the PFK gene family exhibits complex evolutionary characteristics resulting from the combined effects of gene contraction, rapid evolution, and positive selection. In summary, this study reveals the genomic evolutionary basis for the glucose metabolic adaptation of the Chinese longsnout catfish and identifies the PFK gene family as a key candidate for elucidating its unique glucose metabolic characteristics.

## Simple Summary

Glucose metabolism is crucial for animal growth and energy utilization. The Chinese longsnout catfish (*Leiocassis longirostris*) is a typical carnivorous fish with a relatively poor ability to utilize glucose. However, its genetic adaptation mechanisms remain unclear. This study systematically analyzed the evolutionary characteristics of genes related to glucose metabolism in the Chinese longsnout catfish. Some key metabolic genes were subject to rapid evolution and positive selection; among them, the PFK gene family underwent contraction and exhibited characteristics of rapid evolution and positive selection. This study provides new insights into the genetic basis of glucose metabolic adaptation in the Chinese longsnout catfish.

## 1. Introduction

Teleost fish are generally considered glucose intolerant and commonly exhibit persistent postprandial hyperglycemia in comparison with terrestrial mammals [1,2]. However, the degree of glucose intolerance varies considerably among different teleost lineages, suggesting that glucose metabolism evolved in distinct ways [3]. Both herbivorous and omnivorous species can rapidly restore glucose homeostasis [4,5], indicating a relatively strong capacity for glucose utilization. Conversely, carnivorous fish exhibit substandard glucose tolerance, which is typified by a significant and sustained increase in blood glucose levels subsequent to the ingestion of glucose or high-carbohydrate sustenance [6,7,8]. The marked divergence in glycemic responses among fish species suggests that glucose metabolic regulation has undergone evolutionary diversification across teleost lineages [9]. Nonetheless, the genetic and molecular mechanisms underlying these metabolic adaptations are not yet fully elucidated.

In mammals, impaired glucose tolerance is often associated with defects in insulin secretion or insulin signaling [10]. However, recent studies indicate that insulin responses in teleost fish are largely conserved [1], suggesting that impaired glucose tolerance in carnivorous fish may arise from downstream metabolic regulation rather than defective insulin signaling. Glycolysis and gluconeogenesis are central metabolic pathways regulating glucose utilization and production, respectively [11,12,13]. The balance between these pathways influences the capacity of organisms to process exogenous glucose and may represent an important factor shaping glucose metabolic adaptation among fish species. Previous studies have shown that herbivorous fish rapidly induce the expression of key glycolytic enzymes after feeding. In contrast, carnivorous fish exhibit a limited glycolytic response to carbohydrate loading [3,14]. In addition, hepatic gluconeogenesis in carnivorous fish is not effectively suppressed after high carbohydrate intake, resulting in continued endogenous glucose production [14,15,16]. Recent comparative genomic studies have demonstrated that adaptive evolution of metabolic pathways is closely associated with gene family expansion, contraction, and positive selection [17,18]. In particular, genes that encode rate-limiting enzymes frequently occupy key regulatory positions within metabolic networks. Alterations in their sequences and functions can considerably influence metabolic flux, thereby rendering them more vulnerable to natural selection during evolutionary adaptation [19,20]. Glycolysis and gluconeogenesis comprise multiple rate-limiting enzymes, and adaptive evolution of the genes encoding these enzymes may contribute to divergent glucose metabolic capacities among fish species. However, which rate-limiting enzyme-encoding genes are associated with glucose metabolic adaptation and the evolutionary mechanisms shaping these genes remain largely unexplored.

Chinese longsnout catfish (*Leiocassis longirostris*) belongs to the order *Siluriformes* and the family *Bagridae*, and is a high-value economic freshwater fish in China [21,22,23]. Chinese longsnout catfish is a typical benthic carnivorous fish [24]. Due to its long-term adaptation to a low-carbohydrate dietary environment, Chinese longsnout catfish exhibits limited utilization of dietary carbohydrates. This adaptation is characterized by persistent hyperglycemia and hepatic lipid accumulation following high-carbohydrate feeding [5,25]. However, the genetic basis and underlying evolutionary mechanisms associated with glucose metabolic adaptation in carnivorous teleosts, such as the Chinese longsnout catfish, remain largely unexplored. To address this, this study constructed a multispecies comparative framework spanning key evolutionary nodes, from cartilaginous fish outgroups to bony fishes with diverse dietary preferences. We then focused on glucose metabolic adaptation in the Chinese longsnout catfish and systematically analyzed the evolutionary patterns of genes involved in glycolysis and gluconeogenesis. In this study, gene family analyses, phylogenetic reconstruction, and selection pressure assessments (including branch and branch-site models) were conducted to identify candidate genes associated with glucose metabolic adaptation and characterize their evolutionary patterns. In addition, gene structure analysis, conserved domain annotation, protein motif identification, and interspecies synteny analysis were integrated to further investigate the structural characteristics of the candidate gene family. Overall, this study provides genomic insights into the genetic basis of glucose metabolism adaptations in teleosts.

## 2. Materials and Methods

### 2.1. Cluster Analysis of Gene Families

In this study, high-quality whole-genome assemblies, annotation files, protein sequences, and coding sequences of seven representative vertebrate species were retrieved from the NCBI public database (https://www.ncbi.nlm.nih.gov/) (Table S1). The selection of seven representative jawed vertebrates were selected based on phylogenetic representativeness (teleosts, basal ray-finned fishes, and cartilaginous outgroups), dietary diversity (carnivores, herbivores, and omnivores), and chromosome-level genome quality. The dataset under consideration included two species of herbivorous teleosts: grass carp (*Ctenopharyngodon idella*, GCF_019924925.1) and blunt snout bream (*Megalobrama amblycephala*, GCF_018812025.1). The dataset also included two species of carnivorous teleosts: Chinese longsnout catfish (genome from a previous study [24]) and yellow catfish (*Tachysurus fulvidraco*, GCF_022655615.1). Zebrafish (*Danio rerio*, GCF_049306965.1) were selected as the model teleosts. The sterlet (*Acipenser ruthenus*, GCF_902713425.1), an early-diverging actinopterygian, was included for comparative analysis. The elephant shark (*Callorhinchus milii*, GCF_018977255.1), a cartilaginous fish, was selected as the outgroup. To identify orthologous genes across species, redundant protein isoforms were first filtered to retain only the longest transcript for each gene. Subsequently, all protein sequences were subjected to all-versus-all BLASTP (v2.12.0) searches (*E*-value ≤ 1 × 10^−5^), and high-throughput clustering analysis was performed using OrthoFinder (v3.1.2) [26]. Ultimately, a total of 1493 strict one-to-one orthologous genes (single-copy orthologs) were identified and retained for downstream phylogenetic reconstruction and divergence time estimation across all seven species.

### 2.2. Phylogenetic Reconstruction and Divergence Time Estimation

For the 1493 identified single-copy orthologous genes, multiple sequence alignments of protein sequences were performed using MAFFT (v7.505) for each gene independently [27]. Poorly aligned positions and regions with low conservation were subsequently removed using TrimAl (v1.5) [28]. After trimming, all 1493 individual ortholog alignments were concatenated into a single supermatrix using the AMAS.py script, thereby generating a genome-scale dataset for phylogenetic reconstruction [29]. A maximum-likelihood species tree was constructed using IQ-TREE v2.2.0, with branch support evaluated by 1000 ultrafast bootstrap and SH-aLRT replicates [30]. To estimate divergence times among species, a Bayesian relaxed molecular clock approach was implemented in the MCMCTREE program within the PAML (v4.10.10) package [31]. Time calibrations were derived from the TimeTree database (http://www.timetree.org/) and relevant fossil records. Three strict fossil calibration constraints were applied: zebrafish–elephant shark (440.8–495.2 million years ago (Mya)), zebrafish–sterlet (333.4–372.0 Mya), and zebrafish–Chinese longsnout catfish (132.0–170.0 Mya). Two independent MCMC runs were conducted to ensure convergence. Each run included a burn-in of 2000 iterations, followed by sampling every 10 steps, yielding a total of 20,000 posterior samples for parameter estimation.

### 2.3. Gene Family Expansion and Contraction

Gene family expansion and contraction refer to significant changes in the number of genes within a gene family among one or more species and provide important insights into adaptive genome evolution. To identify lineage-specific gene family evolution, gene family expansion and contraction analyses were performed using CAFE5 (v4.2.1) [32]. The analysis was based on the gene count matrix of multi-copy orthologous gene families (orthogroups) generated by OrthoFinder and the ultrametric species tree with absolute divergence times inferred by MCMCTREE. Gene families exhibiting significant lineage-specific expansion or contraction were identified using a significance threshold of *p* < 0.05. To investigate the evolutionary dynamics of genes involved in glucose metabolism, particularly the glycolysis and gluconeogenesis pathways, the copy numbers of key rate-limiting enzyme genes were extracted and quantified from the OrthoFinder-derived orthogroups. The copy numbers of key glucose metabolism-related genes were compared among representative fish species to identify lineage-specific patterns of gene retention and loss.

### 2.4. KEGG Enrichment Analysis

To investigate the potential biological functions associated with lineage-specific gene family expansion and contraction, genes belonging to significantly expanded or contracted gene families identified by CAFE5 were subjected to Kyoto Encyclopedia of Genes and Genomes (KEGG) pathway enrichment analysis. The corresponding KEGG Orthology (KO) annotations of these genes were obtained from the genome functional annotation results. The KO identifiers of expanded or contracted genes were extracted and used as input for enrichment analysis using the clusterProfiler R package (v4.10.1) [33]. KEGG enrichment analysis was performed against the KO database (organism = “ko”) using a hypergeometric test [34]. Multiple testing correction was performed using the Benjamini–Hochberg method, and pathways with an *p* adj < 0.05 were considered significantly enriched. The enriched KEGG pathways were visualized using dot plots generated by the clusterProfiler package. The set of all genes with assigned KO annotations in the corresponding genome was used as the background gene set for enrichment analysis.

### 2.5. Analysis of Rapidly Evolving Genes

To identify genes with accelerated evolutionary rates in the glycolysis and gluconeogenesis pathways in the Chinese longsnout catfish lineage, rapid evolution analyses were performed on all genes associated with these pathways. First, the coding sequences (CDSs) of each target gene were extracted from all examined species. Before performing a multiple sequence alignment, the remove_stop_codons.py script was used to remove terminal stop codons from all CDSss. The processed CDSs were aligned using MACSE (v2.07) [35], and the resulting alignments were converted to the PHYLIP format required by PAML with the convert_to_paml_phy.py script. For each orthogroup within the glycolysis and gluconeogenesis pathways, an individual gene tree was reconstructed. In each analysis, the branch corresponding to Chinese longsnout catfish was designated as the foreground branch and labeled with #1, whereas all remaining branches were treated as background branches. Rapidly evolving genes (REGs) were identified using the branch model implemented in the CODEML program of the PAML package [31]. Specifically, the two-ratio model (model = 2, NS sites = 0) was used to estimate the ω (*d*N/*d*S) ratio of the foreground branch independently from the background branches. This model was compared with the one-ratio null model (model = 0, NS sites = 0), which assumes a single ω ratio across all branches, using a likelihood ratio test (LRT). To account for multiple testing across genes, *p*-values obtained from the LRTs were adjusted using the Benjamini–Hochberg false discovery rate (FDR) procedure. Genes were defined as rapidly evolving genes when the *p* adj < 0.05 and the estimated ω value of the foreground branch exceeded that of the background branches.

### 2.6. Positive Selection Analysis

Positive selection analyses were conducted to identify genes and amino acid substitutions under positive selection in the glycolysis and gluconeogenesis pathways. The codon alignments and gene tree topologies described above were used for these analyses. The branch-site model (Model A; model = 2, NSsites = 2) implemented in the CODEML program of the PAML package was applied, allowing a subset of codon sites along the foreground branch to evolve under positive selection (ω > 1) [31]. Model A was compared with the corresponding null model, in which the ω value of the foreground branch was constrained to be no greater than 1. *p*-values derived from the LRTs were adjusted using the Benjamini–Hochberg FDR method. Genes showing significant evidence of positive selection (*p* adj < 0.05) were further examined using the Bayes Empirical Bayes (BEB) method to estimate the posterior probabilities of individual codon sites under positive selection along the foreground branch [36]. Amino acid sites with a posterior probability greater than 0.95 were considered candidate positively selected sites and were defined as key adaptive substitutions in the corresponding genes.

### 2.7. Multiple Sequence Alignment, Structural Localization of Positive Selection Sites, and 3D Visualization

Using ESPRIPT 3.2, we generated multiple sequence alignment diagrams for PFK (phosphofructokinase) family proteins from seven fish species to evaluate the conservation of positive selection sites and their flanking sequences. To clarify the distribution of positively selected sites in the PFKP (PFK, platelet type) of the Chinese longsnout catfish within its three-dimensional structure and their functional implications, this study performed structural localization of the BEB sites predicted by the branch-site model. Since the site numbers output by PAML are based on a multi-sequence codon matrix that includes alignment gaps, coordinate recalibration is required to eliminate site shifts caused by gaps and alignment trimming. First, a global sequence alignment and coordinate restoration were performed between the codon-aligned sequences of the foreground branch and the full-length amino acid sequence of the long-snouted loach PFKP. Subsequently, based on the full-length amino acid sequence of the long-snouted loach PFKP, AlphaFold3 (AlphaFold Server: https://alphafoldserver.com/) was used to predict the full-length three-dimensional structural model of its monomer. Additionally, to define domain boundaries and functional contexts, UniProtKB (https://www.uniprot.org/) (Accession ID: Q01813) and its Feature Viewer tool were used to map domain regions (N-terminal catalytic domain: residues 1–399; interdomain linker: 400–411; C-terminal regulatory domain: 412–784) and retrieve functional site annotations.

### 2.8. Comparative Synteny Analysis

To investigate genome-wide syntenic relationships among Chinese longsnout catfish, grass carp, and zebrafish, comparative whole-genome synteny analysis was performed using TBtools (v2.485) [37]. Genome assemblies, together with their corresponding gene annotation files (GFF3/GTF format), were used as input. Orthologous syntenic blocks were identified using the One Step MCScanX-Super Fast module implemented in TBtools, with an *E*-value cutoff of 1 × 10^−5^ [38]. The identified syntenic blocks were subsequently visualized using the Multiple Synteny Plot module to generate whole-genome synteny maps.

### 2.9. Exon–Intron Organization, Motif, and Conserved Domain Analysis of the PFK Family

To characterize the structural conservation of the PFK gene family, codon-aware multiple sequence alignments were performed on the CDSs of all PFK genes identified from the seven species using MACSE (v2.06) [35]. Corresponding amino acid alignments were subsequently generated to ensure accurate alignment in the presence of potential frameshift mutations or premature stop codons. Conserved protein motifs were predicted using the MEME Suite (v5.5.9) online server, with the maximum number of motifs set to 10 while all other parameters were kept at their default values [39]. Conserved protein domains were identified using the NCBI Batch CD-Search tool (https://ncbi.nlm.nih.gov/Structure/bwrpsb/bwrpsb.cgi, accessed on 24 August 2026). Finally, phylogenetic relationships, conserved motif distributions, gene structures, and protein domain architectures were integrated and visualized using the Gene Structure View (Advanced) module implemented in TBtools [37].

## 3. Results

### 3.1. Phylogenetic Relationships and Evolutionary Timeline

**Figure 1:**
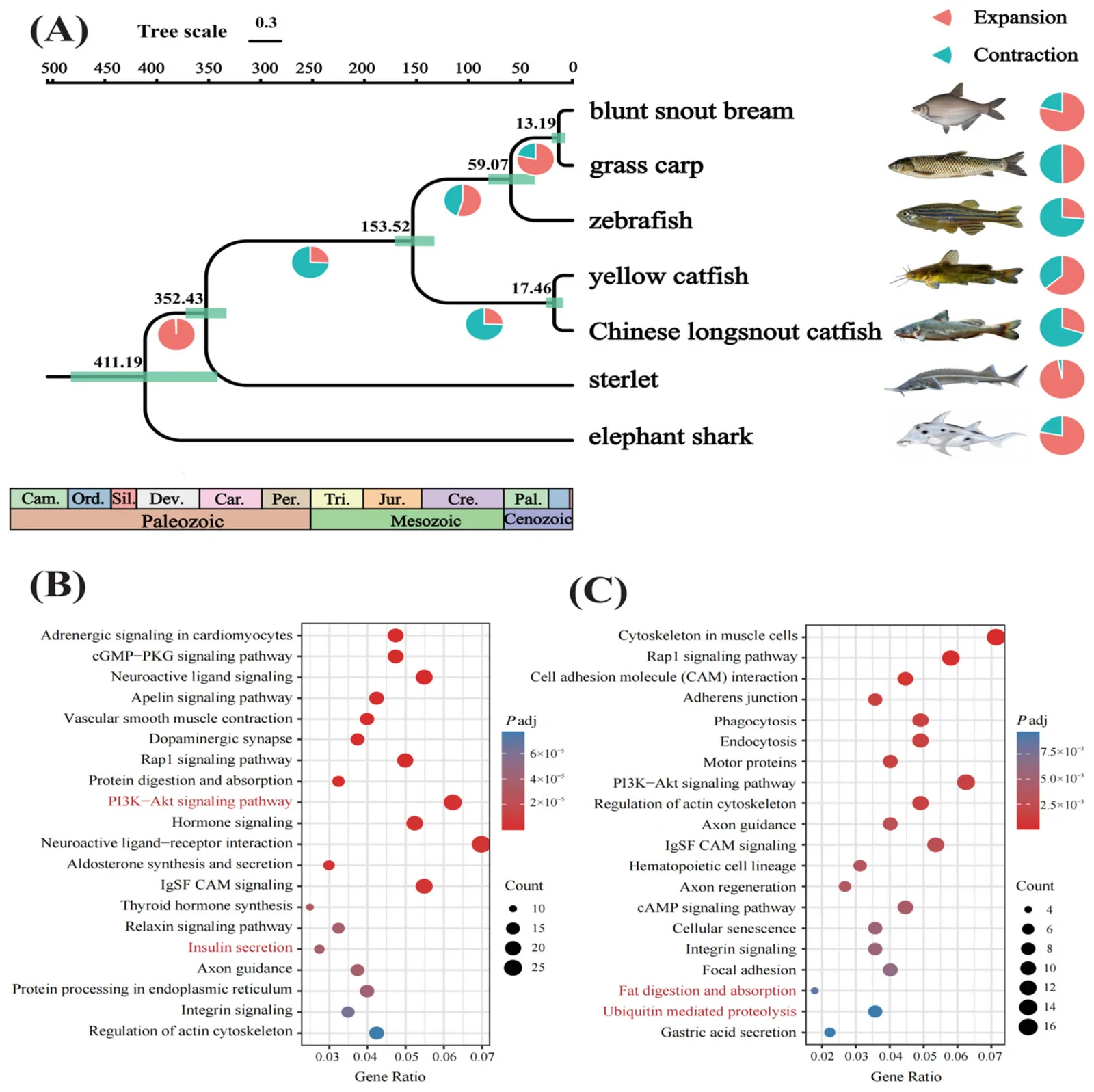
Phylogenetic relationships and gene family expansion and contraction analyses among seven fish species. (**A**) Phylogenetic tree constructed for seven fish species, showing divergence times and proportions of expanded and contracted gene families across lineages. Cam, Cambrian; Ord, Ordovician; Sil, Silurian; Dev, Devonian; Car, Carboniferous; Per, Permian; Tri, Triassic; Jur, Jurassic; Cre, Cretaceous; Pal, Paleogene. (**B**) KEGG enrichment analysis of contracted gene families identified in the branch leading to Chinese longsnout catfish and yellow catfish. (**C**) KEGG enrichment analysis of expanded gene families identified in the branch leading to Chinese longsnout catfish and yellow catfish. The KEGG pathways highlighted in red represent the key pathways discussed in this study.

A time-calibrated phylogenetic tree was reconstructed using genome-wide data from seven representative vertebrate species. The phylogenetic analysis showed that the Cyprinidae fish, including blunt snout bream, grass carp, and zebrafish, formed a well-supported clade, which diverged from the *Siluriformes* lineage containing yellow catfish and Chinese longsnout catfish approximately 153.52 Mya (Figure 1A). The divergence times between grass carp and blunt snout bream, and between Chinese longsnout catfish and yellow catfish, were estimated at approximately 13.19 Mya and 17.46 Mya, respectively.

### 3.2. Gene Family Expansion and Contraction Analysis

Genome-wide gene family evolution analysis revealed lineage-specific patterns of gene family expansion and contraction among the examined fish. Grass carp and blunt snout bream exhibited significant expansions of gene families along their respective evolutionary lineages, whereas Chinese longsnout catfish showed a relatively higher proportion of contracted gene families. To further investigate the evolutionary significance of gene family changes in the *Siluriform* lineage, KEGG enrichment analysis was performed on contracted and expanded gene families identified in the branch leading to Chinese longsnout catfish and yellow catfish. The results revealed significant contraction of glucose metabolism-related pathways (Figure 1B), indicating lineage-specific alterations in the genomic composition of carbohydrate metabolism-related genes. Among the contracted pathways, carbohydrate digestion and absorption showed a marked reduction, indicating potential changes in gene families involved in dietary carbohydrate processing and glucose transport. In addition, significant contraction of the insulin signaling pathway and its downstream PI3K-Akt signaling pathway suggested evolutionary remodeling of insulin-mediated glucose uptake and intracellular metabolic regulation. Furthermore, contraction of the glucagon signaling pathway indicated potential alterations in endocrine regulation associated with glucose homeostasis.

In contrast, pathways related to protein and lipid metabolism, including ubiquitin-mediated proteolysis and fat digestion and absorption, showed expansion patterns (Figure 1C). These evolutionary signatures suggest that Chinese longsnout catfish may have undergone metabolic specialization toward preferential utilization of dietary proteins and lipids rather than carbohydrates, consistent with its carnivorous feeding strategy.

### 3.3. Copy Number Evolution of Glucose Metabolism-Related Genes

**Figure 2:**
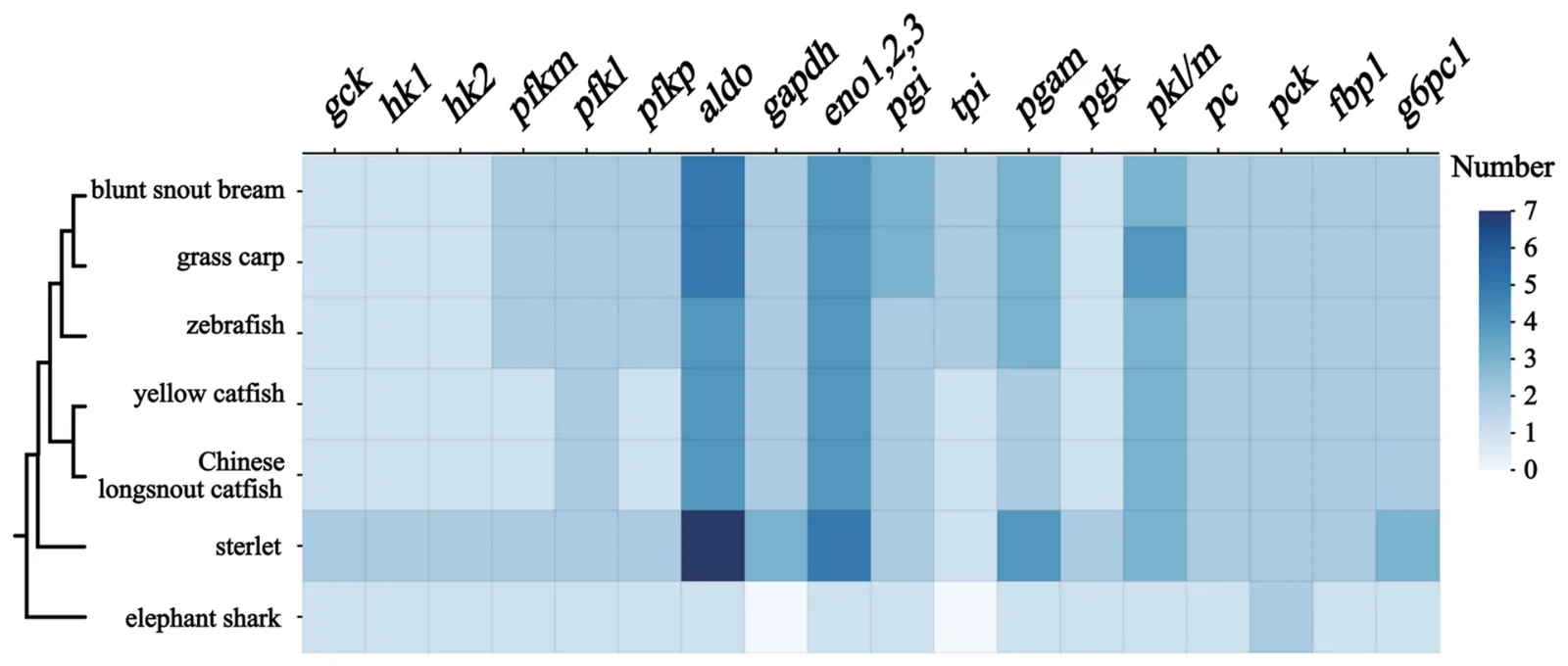
Heatmap showing copy numbers of key genes involved in glycolysis and gluconeogenesis across seven fish species. Gene abbreviations in the heatmap: *gck*, glucokinase; *hk1/2*, hexokinase 1/2; *pfkm/l/p*, phosphofructokinase, muscle/liver/platelet; *aldo*, fructose-bisphosphate aldolase; *gapdh*, glyceraldehyde-3-phosphate dehydrogenase; *eno1,2,3*, α/β/γ-enolase; *pgi*, phosphoglucose isomerase; *tpi*, triosephosphate isomerase; *pgam*, phosphoglycerate mutase; *pgk*, phosphoglycerate kinase; *pkl/m*, pyruvate kinase, liver/muscle; *pc*, pyruvate carboxylase; *pck*, phosphoenolpyruvate carboxykinase; *fbp1*, fructose-bisphosphatase 1; *g6pc1*, glucose-6-phosphatase catalytic subunit 1.

Copy number heatmap analysis of 18 core genes in the glycolysis and gluconeogenesis pathways further revealed metabolic adaptation patterns among fish with different feeding habits (Figure 2). As an outgroup, elephant shark exhibited predominantly single-copy or lost genes, with complete absence of several genes such as glyceraldehyde-3-phosphate dehydrogenase (*gapdh*) and triosephosphate isomerase (*tpi*). In contrast, sterlet displayed gene-specific expansions likely associated with its lineage-specific whole-genome duplication, including increased copy numbers of fructose-bisphosphate aldolase (*aldo*) genes (up to seven copies). Among teleosts, the herbivorous species (grass carp and blunt snout bream) retained multiple copies of key glycolytic genes, including *pfkm*, *pfkp*, and α/β/γ-enolase (*eno1/2/3*) (2–3 copies), while *aldo* expanded to five copies. In comparison, carnivorous fish (Chinese longsnout catfish and yellow catfish) exhibited contraction of several key glycolytic genes, including *pfkm*, *pfkp*, and *tpi*, each generally retaining only a single copy. Overall, expansion and retention of glycolytic genes in herbivorous fish are consistent with their higher capacity for carbohydrate utilization. Conversely, the diminished copy numbers observed in Chinese longsnout catfish may contribute to its glucose intolerance and carnivorous metabolic specialization at the genomic level.

### 3.4. Identification of REGs in Glucose Metabolism Pathway

An investigation was conducted to ascertain the evolutionary characteristics of glucose metabolism-related genes in Chinese longsnout catfish. To this end, selection pressure analyses were conducted on key enzymes in the glycolysis and gluconeogenesis pathways using the branch model implemented in PAML (Table 1). The two-ratio model, which designated Chinese longsnout catfish as the foreground branch, provided a significantly better fit than the one-ratio model for five genes, indicating accelerated evolution in Chinese longsnout catfish.

Specifically, in the glycolysis pathway, the hexokinase 2 (*hk2*), which catalyzes the first step of glucose metabolism, exhibited a strong signal of accelerated evolution (2ΔlnL = 106.028, *p* < 0.001). The key rate-limiting enzyme gene *pfkm* showed significant evidence of lineage-specific accelerated evolution (2ΔlnL = 5.796, *p* = 0.016). Its ω value was higher in the Chinese longsnout catfish branch (ω_1_ = 0.325) than in the background branches (ω_0_ = 0.061). In the gluconeogenesis pathway, multiple key enzymes similarly exhibited accelerated evolution signals. The phosphoenolpyruvate carboxykinase (*pck*) showed a pronounced signature (2ΔlnL = 16.900, *p* < 0.001), with a markedly elevated ω value in the foreground branch (ω_1_ = 0.686; background ω_0_ = 0.073). Furthermore, the analysis revealed that pyruvate carboxylase (*pc*) (2ΔlnL = 4.349, *p* = 0.037) and glucose-6-phosphatase (*g6pc*) (2ΔlnL = 8.737, *p* = 0.003) also showed significant signals of rate acceleration.

Overall, key enzymes involved in glycolysis and gluconeogenesis exhibited lineage-specific rate acceleration in Chinese longsnout catfish. These findings suggest that core glucose metabolism components have undergone adaptive sequence evolution. This evolutionary pattern may contribute to the species-specific metabolic adaptation to glucose intolerance.

### 3.5. Positive Selection and Adaptive Sites in Glucose Metabolism Genes

To further investigate positive selection and site-specific adaptive evolution of key genes in the glycolysis and gluconeogenesis pathways in Chinese longsnout catfish, branch-site model analyses were conducted using PAML (Table 2). LRTs indicated that the alternative model (Model A) provided a significantly better fit than the null model (Model A0) for *pfkp* (2ΔlnL = 36.558, *p* < 0.001), *pkm* (2ΔlnL = 19.410, *p* < 0.001), *pck* (2ΔlnL = 51.546, *p* < 0.001), *hk2* (2ΔlnL = 166.684, *p* < 0.001), and *gapdh* (2ΔlnL = 7.203, *p* < 0.001), indicating strong evidence of positive selection acting on these genes in the Chinese longsnout catfish lineage.

BEB analysis identified multiple positively selected sites with high posterior probabilities (>0.95) in these genes. In particular, *hk2* contained numerous positively selected codons, including sites 126W, 127G, and 132C, among others. *pfkp* exhibited three positively selected sites (250S, 266G, and 272S), while single or limited positively selected sites were detected in *pkm* (site 216T) and *pck* (site 4C). No sites with substantial posterior probability were identified in *gapdh*.

**Figure 3:**
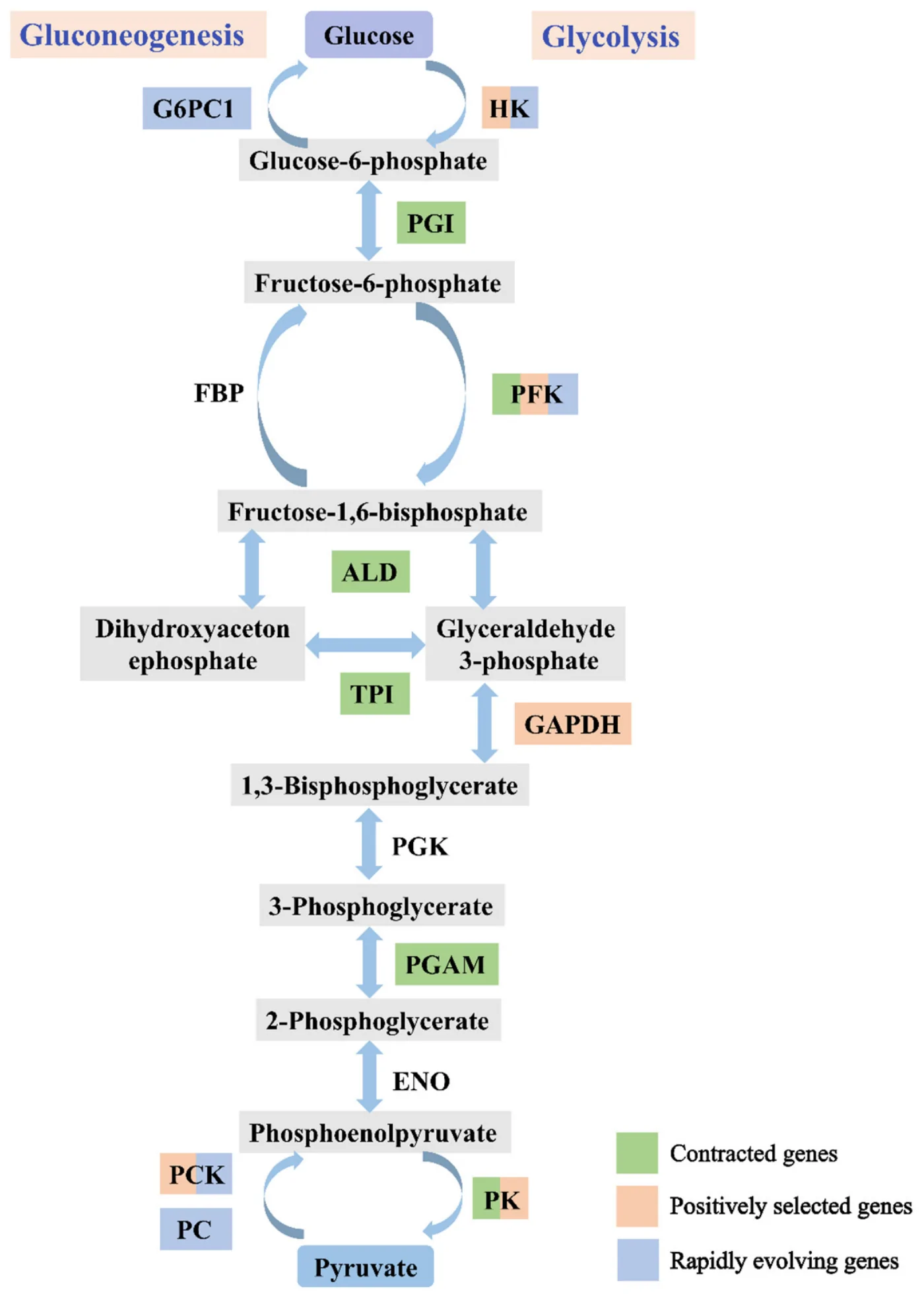
Evolutionary signatures of key glycolysis (right) and gluconeogenesis (left) genes in Chinese longsnout catfish. Gene abbreviations are consistent with Figure 2.

These results indicate that key rate-limiting enzymes in the glycolysis and gluconeogenesis pathways of Chinese longsnout catfish exhibit lineage-specific accelerated evolution accompanied by positively selected amino acid substitutions. Notably, among all analyzed metabolic genes, PFK was the only rate-limiting enzyme exhibiting a combination of gene family contraction, positive selection, and rapid evolution (Figure 3). This unique pattern, characterized by reduced gene copy number but accelerated evolution of the retained copy under positive selection, highlights PFK as a key candidate gene family for further investigation of glucose metabolic adaptation in Chinese longsnout catfish.

**Figure 4:**
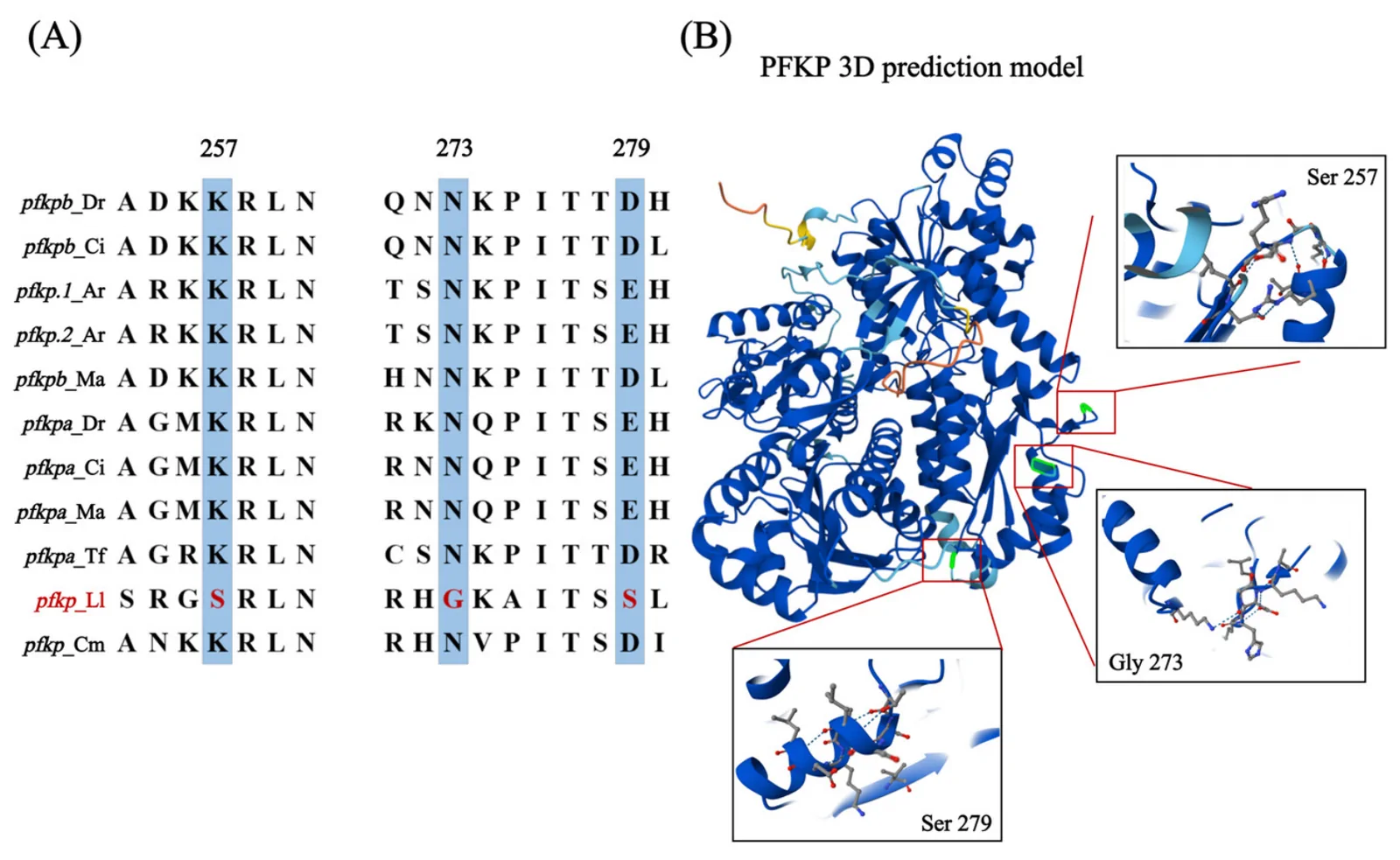
Sequence alignment and 3D structural mapping of positively selected sites in PFKP. (**A**) Multiple sequence alignment of PFK family proteins across representative teleost species surrounding the positively selected sites. The foreground branch (*pfkp*_Ll) is highlighted in red, with its specific amino acid residues marked in red. Ll: *Leiocassis longirostris*; Ci: *Ctenopharyngodon Idella*; Ma: *Megalobrama amblycephala*; Tf: *Tachysurus fulvidraco*; Dr: *Danio rerio*; Ar: *Acipenser ruthenus*; Cm: *Callorhinchus milii*. (**B**) Predicted 3D tertiary structure of *L. longirostris* PFKP generated by AlphaFold3 (pLDDT scores: dark blue, very high confidence; light blue, confident; yellow/orange, low confidence). The three mapped positively selected sites are highlighted in bright green. Insets display close-up views of the Ser257, Gly273, and Ser279, respectively, represented as stick models. *pfkp*, phosphofructokinase, platelet.

To elucidate the structural and functional consequences of adaptive evolution in PFKP, positively selected sites identified by the branch-site model were mapped onto its tertiary structure. To resolve coordinate shifts caused by alignment gaps in the multi-species codon matrix, pairwise sequence alignment was performed against the native full-length amino acid sequence of Chinese longsnout catfish PFKP, accurately calibrating the BEB sites (250S, 266G, and 272S) to native residues Ser257, Gly273, and Ser279, respectively (Figure 4A). The 3D structure of Chinese longsnout catfish PFKP predicted by AlphaFold3 exhibited exceptional overall global topological reliability, with a predicted template modeling (pTM) score of 0.95 (Figure 4B). Crucially, all three positively selected sites were located within very high confidence structural regions (pLDDT > 90, indicated in dark blue). Domain mapping shows that residues 257, 273, and 279 are all located entirely within the N-terminal catalytic domain of PFKP (residues1–399). Functional site annotations indicate that residue 273 is involved in binding the substrate from the other chain, confirming its location at a critical subunit interface for oligomerization; spatial distance measurements and surface topology analysis indicate that sites 257 and 279 are relatively far from site 273 (17.1 Å and 22.9 Å, respectively) and are located in three mutually non-connected surface grooves (Figure S1). These features suggest that the three sites do not form a single binding pocket. Analysis of the local chemical environment reveals that Ser257 and Ser279 form a local network of polar interactions and hydrogen bonds with neighboring residues, whereas Gly273 is located in a flexible loop region lacking a defined secondary structure (Figure 4B).

### 3.6. Phylogenetic and Syntenic Analyses of the PFK Gene Family

**Figure 5:**
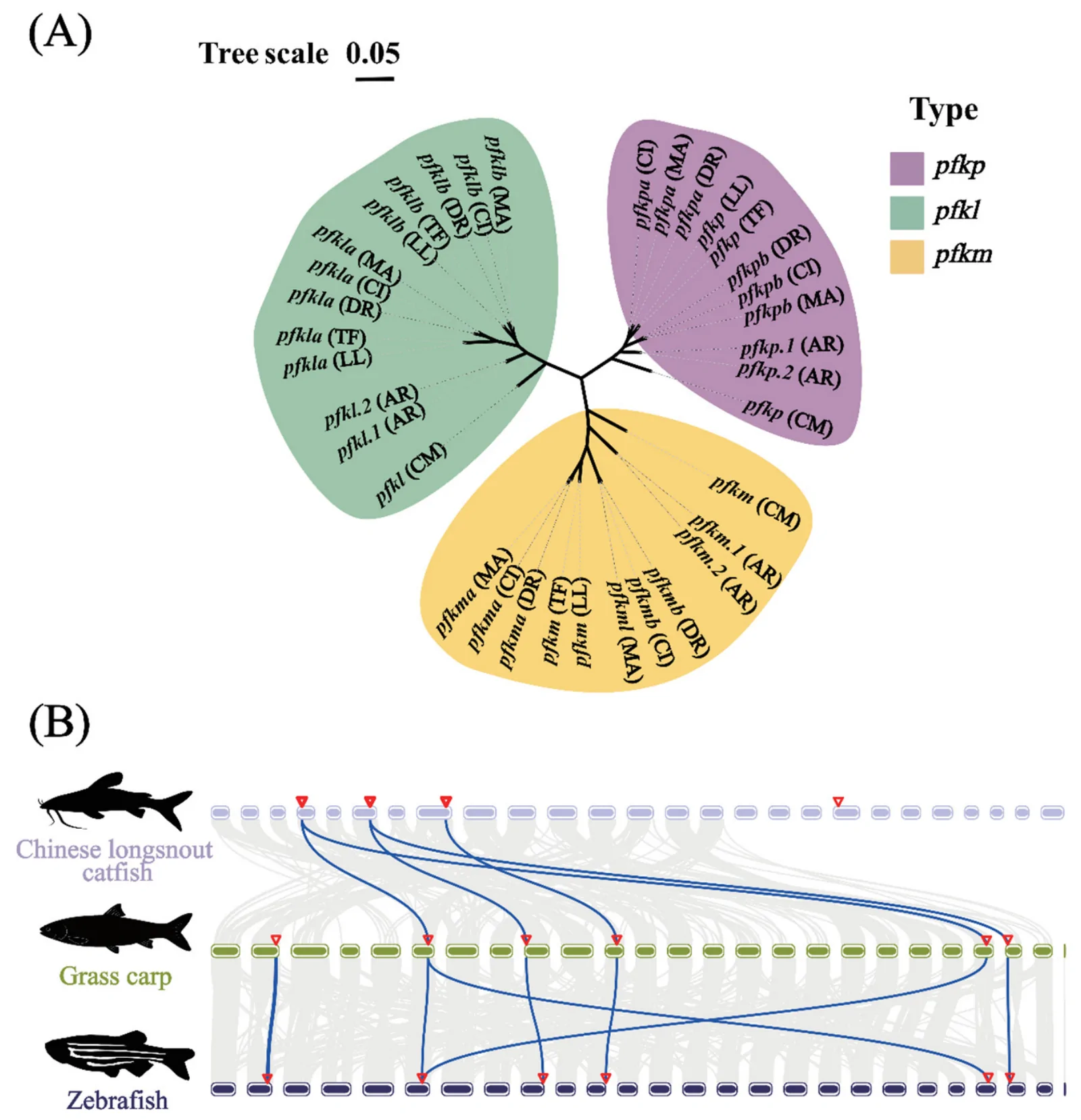
Phylogenetic analysis and interspecies synteny analysis of the PFK gene family. (**A**) Phylogenetic tree of the PFK gene family, including *pfkl*, *pfkm*, and *pfkp*. *pfkm/l/p*, phosphofructokinase, muscle/liver/platelet. Species abbreviations are as defined in Figure 4. (**B**) Comparative genomic synteny analysis among zebrafish, grass carp, and Chinese longsnout catfish. Red ▼ indicate members of the PFK gene family. Blue lines indicate syntenic relationships within the PFK gene family. Grey lines indicate whole-genome syntenic gene pairs.

Phylogenetic and microsynteny analyses revealed that the PFK gene family cleanly resolved into three distinct clades: *pfkp*, *pfkl*, and *pfkm* (Figure 5A). Within the teleost lineage, these subfamilies underwent further diversification driven by the teleost-specific genome duplication event (TGD). Specifically, the *pfkl* branch underwent a divergence, resulting in the establishment of two paralogous subfamilies: *pfkla* and *pfklb*. The Chinese longsnout catfish and the representative cyprinids (grass carp and zebrafish) retained both paralogs, with their corresponding chromosomal loci exhibiting highly conserved microsyntenic blocks (Figure 5B). Conversely, comparative genomic analysis revealed lineage-specific copy number changes in other PFK subtypes. In Chinese longsnout catfish, *pfkm* and *pfkp* were retained as single-copy genes, whereas *pfkl* maintained two copies. Synteny analysis revealed that the chromosomal regions containing *pfkm* and *pfkl* displayed highly conserved syntenic relationships among the three species, indicating strong conservation of these gene loci across teleost lineages. However, the *pfkp* exhibited pronounced lineage-specific genomic variation. Although *pfkp* maintained conserved syntenic relationships between grass carp and zebrafish, the *pfkp* locus in Chinese longsnout catfish was located in a genomic region lacking corresponding syntenic connections, suggesting that this gene may have undergone lineage-specific genomic rearrangement or locus repositioning.

Collectively, the evolutionary history of the PFK gene family in the Chinese longsnout catfish lineage was characterized not only by asymmetric loss of TGD-derived paralogs but also by lineage-specific repositioning of the *pfkp* locus. The loss of syntenic conservation and reduction in copy number of *pfkp* may reflect adaptive genomic remodeling associated with glucose metabolism in Chinese longsnout catfish and may contribute to its distinctive glucose intolerance phenotype.

### 3.7. Structural Features of PFK Family Genes

**Figure 6:**
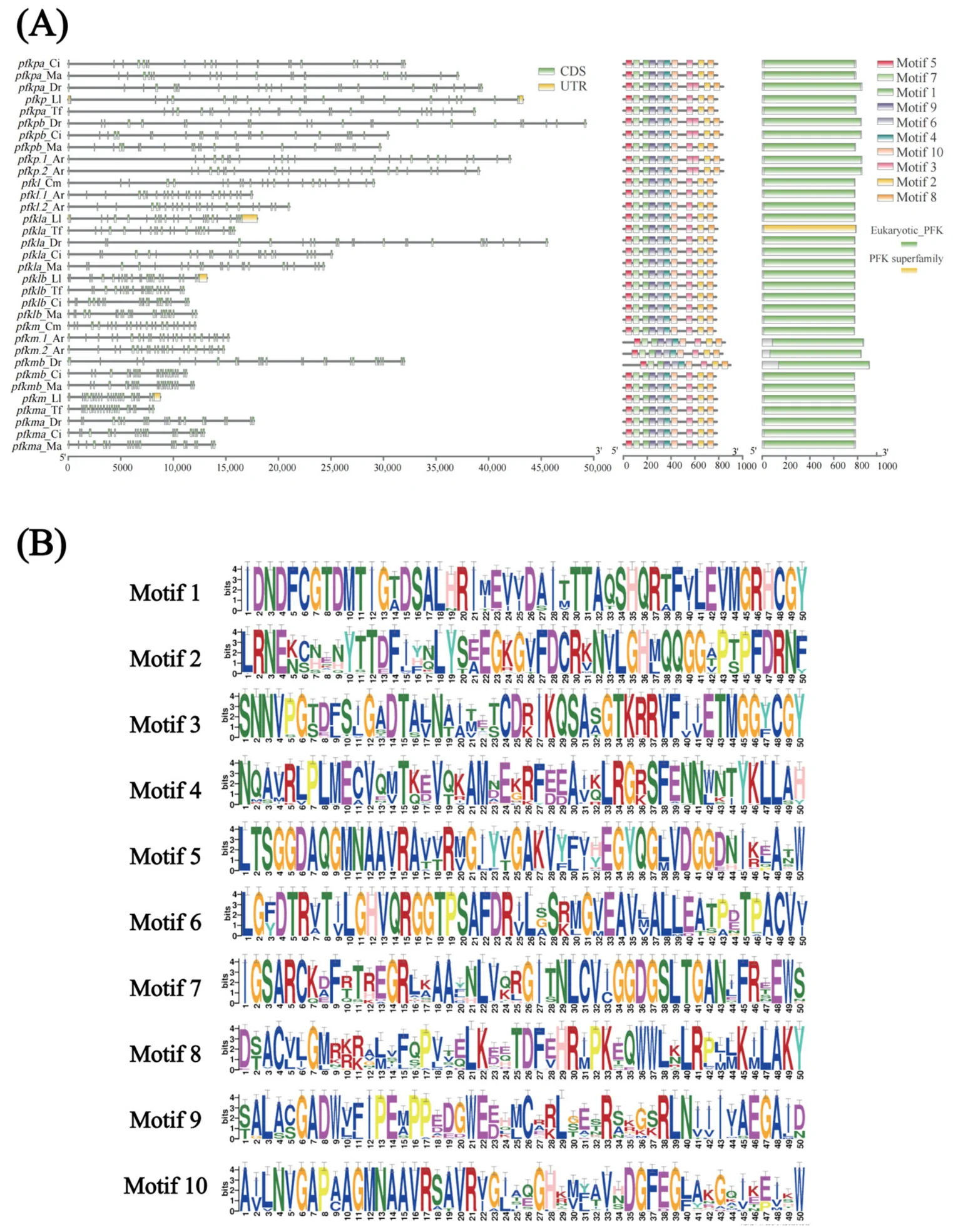
Structural characterization of the PFK gene family. (**A**) Comparison of exon–intron organization (left), conserved motifs (middle), and conserved protein domains (right) among PFK family members. (**B**) Sequence conservation of the top 10 protein motifs (Motif 1–10) identified by MEME. *pfkm/l/p*, phosphofructokinase, muscle/liver/platelet. Species abbreviations are as defined in Figure 4.

To further characterize the structural evolution of the PFK gene family in teleosts, we compared the exon–intron organization, conserved motifs, and functional domains of PFK members from representative fish species (Figure 6A). The results demonstrated that all PFK exhibited highly conserved structural features. The ten conserved motifs (Motif 1–10) displayed identical compositions and arrangements across species (Figure 6B), and all PFK contained a complete PFK domain of approximately 800 amino acids. These results suggest that the PFK in Chinese longsnout catfish maintain the typical structural characteristics of the PFK family, without obvious loss or degeneration of conserved domains. At the exon–intron organization level, different PFK subtypes exhibited distinct patterns of genomic organization. The *pfkp* subtype generally exhibited a larger genomic span compared with *pfkl* and *pfkm*. In the *pfkp* subgroup, the genomic length of Chinese longsnout catfish *pfkp* was approximately 43 kb, whereas that of grass carp *pfkpa* was approximately 33 kb. This difference was mainly attributable to variation in intron length. Collectively, these findings suggest that the PFK family has maintained highly conserved protein architectures during teleost evolution. However, different subtypes have experienced lineage-specific changes in genomic organization, which may contribute to their regulatory evolution and adaptive divergence.

## 4. Discussion

This study systematically investigated the evolutionary characteristics of glucose metabolism-related genes in the Chinese longsnout catfish using a comparative genomics approach. The results of this study demonstrate that glucose metabolism-related genes in Chinese longsnout catfish have evolved evolutionary patterns that are distinct from those observed in herbivorous cyprinid fish. The PFK gene family in particular exhibits the most prominent signatures of adaptive evolution.

According to the findings of previous studies, the utilization capacity of glucose is generally limited in carnivorous fish. This limitation is characterized by reduced glycolytic responsiveness, decreased glucose uptake efficiency, and sustained gluconeogenic activity [3,40]. This study revealed a substantial decrease in multiple glucose metabolism-related pathways in catfishes. These pathways encompass carbohydrate digestion and absorption, insulin signaling, and PI3K-Akt signaling. This finding indicates that genomic components implicated in glucose uptake, transport, and metabolic regulation may have undergone a reduction during prolonged evolutionary processes [41,42]. In contrast, the expansion of protein and lipid metabolism-related pathways was concomitant with the nutritional adaptation of catfishes to protein- and lipid-rich food sustenance sources [43,44]. These findings suggest that the glucose metabolic characteristics of the Chinese longsnout catfish may represent a genome-level metabolic adaptation shaped by long-term carnivorous evolution.

Subsequent examination of the evolution of gene copy number revealed distinct patterns of retention of key glycolytic genes among fish species with divergent dietary preferences. A comparative analysis of the copy number variation of key glycolytic genes revealed that herbivorous fish species (grass carp and blunt snout bream) exhibited a retention of relatively higher copy numbers for genes belonging to the *pfkm*, *pfkp*, and *eno* families. In contrast, several glycolytic genes underwent a reduction in copy number in carnivorous fish species (Chinese longsnout catfish and yellow catfish). Previous studies have demonstrated that carnivorous fish generally exhibit weaker glycolytic induction and reduced glucose clearance efficiency after high-carbohydrate feeding. In contrast, herbivorous fish have been shown to possess stronger glucose metabolic regulatory capacity [5,6]. Consequently, variations in glycolytic gene copy retention patterns may be indicative of disparate glucose metabolic strategies that have evolved over time in response to prolonged dietary adaptation [45,46]. Notably, the PFK gene family demonstrated subtype-specific evolutionary patterns. PFK is a core rate-limiting enzyme in the glycolytic pathway, catalyzing the irreversible conversion of fructose-6-phosphate to fructose-1,6-bisphosphate [47]. The PFK gene family mainly comprises three major subtypes: muscle type (*pfkm*), liver type (*pfkl*), and platelet type (*pfkp*) [48]. While *pfkl* demonstrated high levels of conservation across diverse fish lineages, *pfkm* and *pfkp* underwent copy reduction in Chinese longsnout catfish. Since *pfkm* primarily functions in skeletal muscle glycolysis and muscle tissue is an important site of glucose utilization in fish [49], the reduction in *pfkm* copies may be associated with decreased peripheral glucose utilization capacity in Chinese longsnout catfish. These findings suggest that the impaired glucose utilization characteristic of Chinese longsnout catfish is primarily associated with genomic changes affecting peripheral glycolytic capacity rather than with the coordinated degeneration of all glucose metabolic processes. This interpretation is consistent with previous studies proposing that glucose intolerance in carnivorous fish is related to limited peripheral tissue glucose utilization capacity, particularly reduced glycolytic activity and glucose disposal in skeletal muscle [3,6,41].

We further revealed pronounced sequence-level adaptive evolution of key metabolic enzymes involved in glucose metabolism in Chinese longsnout catfish. The branch model and branch-site model analyses identified lineage-specific accelerated evolution and positive selection signals in several key glycolytic and gluconeogenic genes, including *hk2*, *pfkm*, *pfkp*, *pkm*, and *pck*. The results of this study suggest that glucose metabolic adaptation in Chinese longsnout catfish is associated with alterations in gene family composition and adaptive modifications at the protein-coding sequence level. According to the findings of previous studies, genes that encode for pivotal regulatory nodes within metabolic networks are more prone to becoming targets of natural selection, with a particular emphasis on rate-limiting enzymes that regulate metabolic flux [20,50]. Notably, the PFK gene family was the only glycolytic rate-limiting enzyme family to manifest a triad of phenomena: gene family contraction, accelerated evolutionary rate, and positive selection signals. This observation signifies that PFK function as a key evolutionary target during glucose metabolic adaptation in Chinese longsnout catfish. Concurrently, substantial positive selection was identified in *hk2* and *pck*, suggesting adaptive changes in the initial regulatory phase of glycolysis and the key regulatory points of gluconeogenesis. The concurrent evolution of multiple metabolic regulatory nodes suggests that glucose metabolic adaptation in Chinese longsnout catfish was likely shaped by coordinated evolution of the glycolytic and gluconeogenic networks rather than by changes in a single gene.

Subsequent synteny analysis further revealed subtype-specific evolutionary patterns of the PFK gene family in Chinese longsnout catfish. Previous studies have shown that TGD promoted the expansion and diversification of the PFK gene family [51], providing genetic resources for functional retention and adaptive evolution across different fish lineages [52,53]. Following genome duplication events, duplicated genes are often retained through subfunctionalization, neofunctionalization, or selective loss [54,55]. In this study, the *pfkm* and *pfkl* loci exhibited highly conserved syntenic relationships among Chinese longsnout catfish, grass carp, and zebrafish, indicating strong evolutionary constraints on the genomic regions harboring these two subtypes. Specifically, while *pfkm* and *pfkp* experienced selective gene loss resulting in a single-copy state, *pfkl* retained two duplicated paralogs post-TGD in Chinese longsnout catfish. The retention of *pfkl* duplicate copies likely provided the genetic substrate for subfunctionalization or neofunctionalization in hepatic glycolytic regulation. In contrast, although *pfkp* maintained a clear syntenic relationship between grass carp and zebrafish, the corresponding conserved syntenic block was not detected in Chinese longsnout catfish. This suggests that this gene may have undergone lineage-specific genomic rearrangement or positional relocation.

In conjunction with the copy number reduction and positive selection signals identified in *pfkp*, these findings imply that this subtype may have followed an evolutionary trajectory that is distinct from that of *pfkm* and *pfkl* in the Chinese longsnout catfish. Concurrently, the high conservation of PFK protein domains and motifs across fish species suggests that the adaptive evolution of the PFK family did not disrupt its core catalytic architecture. Rather, it is plausible that changes in gene copy, gene structure, and specific amino acid substitutions affecting functional properties may have been involved [56].

In addition to characterizing the genomic architecture and evolutionary dynamics of the PFK gene family in Chinese longsnout catfish, our findings establish this gene family as a critical evolutionary genes shaping divergent glucose tolerance in teleosts. The evolutionary signatures of gene family contraction, accelerated evolution, and positive selection within the PFK family provide a genomic rationale for the diminished glucose metabolic capacity in carnivorous teleosts such as Chinese longsnout catfish. From an applied perspective, these candidate adaptive sites offer a theoretical foundation for optimizing dietary carbohydrate levels and metabolic regulation in carnivorous fish. However, as this study primarily relied on comparative genomics to explore metabolic divergence between Chinese longsnout catfish and herbivorous fishes, functional validation via in vitro enzymatic assays and gene editing remains essential. Furthermore, integrating genomic datasets across a broader diversity of teleosts will be crucial to fully deciphering the genetic architecture underlying dietary carbohydrate utilization.

## 5. Conclusions

This study revealed a multilayered evolutionary pattern underlying glucose metabolic adaptation in Chinese longsnout catfish. This pattern involves changes in glucose metabolism-related pathways, alterations in key gene copy numbers, and adaptive evolution of the PFK gene family. As a central regulatory node of glycolysis, PFK exhibited gene family contraction, rapid evolution, and positive selection signals. This suggests that PFK may represent an important candidate linking genomic evolutionary changes to the glucose metabolic characteristics of Chinese longsnout catfish. Overall, these findings deepen our understanding of the genomic basis underlying glycolytic adaptation in the Chinese longsnout catfish and other carnivorous teleosts, while providing a theoretical foundation for optimizing dietary carbohydrate utilization in aquaculture.

## Figures and Tables

**Table 1:** Branch model analysis of rapidly evolving genes in glycolysis and gluconeogenesis pathways in *Leiocassis longirostris*.

Genes	Foreground Branch	Models	lnL	2ΔlnL	*p* Value	Parameters
*pfkm*	*Leiocassis longirostris*	One-ratio	−31,176.943	5.796	0.016	ω_0_ = 0.062
		Two-ratio	−31,174.045			ω_0_ = 0.061, ω_1_ = 0.325
*pck*	*Leiocassis longirostris*	One-ratio	−9939.627	16.900	<0.001	ω0 = 0.077
		Two-ratio	−9931.177			ω_0_ = 0.073, ω_1_ = 0.686
*pc*	*Leiocassis longirostris*	One-ratio	−13,710.200	4.349	0.037	ω_0_ = 0.044
		Two-ratio	−13,708.026			ω_0_ = 0.042, ω_1_ = 0.096
*hk2*	*Leiocassis longirostris*	One-ratio	−9678.113	106.028	<0.001	ω_0_ = 0.061
		Two-ratio	−9625.099			ω_0_ = 0.042, ω_1_ = 0.349
*g6pc*	*Leiocassis longirostris*	One-ratio	−6948.768	8.737	0.003	ω_0_ = 0.108
		Two-ratio	−6944.400			ω_0_ = 0.104, ω_1_ = 999

Note: lnL, log-likelihood value; 2ΔlnL, twice the log-likelihood difference between alternative and null models; *p* value, statistical significance determined by the likelihood ratio test (LRT); ω_0_ represents the background non-synonymous to synonymous substitution rate ratio, and ω_1_ represents the foreground non-synonymous to synonymous substitution rate ratio. Gene abbreviations are consistent with Figure 2.

**Table 2:** Branch-site model analysis of positively selected genes in glycolysis and gluconeogenesis pathways in *Leiocassis longirostris*.

Genes	Foreground Branch	Models	lnL	2ΔlnL	*p* Value	Parameters	Positively Selected Sites (BEB > 0.95)
*pfkp*	*Leiocassis longirostris*	Ma0	−31,063.411	36.558	<0.001	ω_0_ = 0.059, ω_1_ = 1, ω_2_ = 1	250-0.997, 266-1.000, 272-0.996
		Ma	−31,045.132			ω_0_ = 0.059, ω_1_ = 1, ω_2_ = 977.352	
*pkm*	*Leiocassis longirostris*	Ma0	−8487.876	19.410	<0.001	ω_0_ = 0.055, ω_1_ = 1, ω_2_ = 1	216-0.978
		Ma	−8478.171			ω_0_ = 0.054, ω_1_ = 1, ω_2_ = 999	
*pck*	*Leiocassis longirostris*	Ma0	−9744.573	51.546	<0.001	ω_0_ = 0.034, ω_1_ = 1, ω_2_ = 1	4-0.999
		Ma	−9718.799			ω_0_ = 0.034, ω_1_ = 1, ω_2_ = 999	
*hk2*	*Leiocassis longirostris*	Ma0	−9512.942	166.684	<0.001	ω_0_ = 0.034, ω_1_ = 1, ω_2_ = 1	126-0.999, 127-1.000, 132-1.000, 285-1.000, 291-0.975, 293-0.965, 298-0.972, 299-0.998, 542-0.990, 543-1.000, 544-0.999, 545-0.951, 546-0.991, 547-1.000, 650-0.996, 653-0.992, 655-1.000, 656-0.977, 658-0.999, 660-0.951, 662-0.999, 664-0.999, 666-0.997, 668-0.951, 672-1.000, 675-1.000, 678-0.999, 679-1.000, 682-0.988, 683-0.987, 684-0.959, 685-1.000, 689-1.000, 686-1.000
		Ma	−9429.600			ω_0_ = 0.034, ω_1_ = 1, ω_2_ = 999	
*gapdh*	*Leiocassis longirostris*	Ma0	−4611.929	7.203	<0.001	ω_0_ = 0.037,ω_1_ = 1, ω_2_ = 1	None
		Ma	−4608.328			ω_0_ = 0.037,ω_1_ = 1, ω_2_ = 371.028	

Note: Ma, branch-site model A (alternative model allowing positive selection on the foreground branch); Ma0, null model fixing ω_2_ = 1; ω_0_, ω_1_, and ω_2_ are the *d*N/*d*S ratios for site classes undergoing purifying selection (ω_0_ < 1), neutral evolution (ω_1_ = 1), and positive selection (ω_2_ ≥ 1), respectively; BEB, Bayes Empirical Bayes approach. Positively selected sites identified by BEB (posterior probability > 0.95) are presented in the format of “amino acid position—posterior probability” (e.g., 250-0.997 indicates amino acid position 250 with a posterior probability of 0.997). Definitions for statistical parameters (lnL, 2ΔlnL, *p* value) are the same as in Table 1. Gene abbreviations are consistent with Figure 2.

## Data Availability

The original contributions presented in this study are included in the article. Further inquiries can be directed to the corresponding author.

## Supplementary Materials

The following supporting information can be downloaded at https://www.mdpi.com/article/10.3390/biology15171481/s1. Table S1: Summary of genomic datasets, assembly versions, accession numbers, and dietary classifications for the seven representative fish species analyzed in this study. Figure S1: Spatial distances and surface distribution of positively selected sites in PFKP.
