# Supplementary material for: Adaptive Evolution of the PFK Gene Family in Chinese Longsnout Catfish, Leiocassis longirostris
Source: Biology (Basel). 2026 Sep 1;15(17):1481. doi: 10.3390/biology15171481 (PMC13564638; doi:10.3390/biology15171481)
Supplement: Supplementary file 1 [file biology-15-01481-s001.zip › Figure S1.pdf]

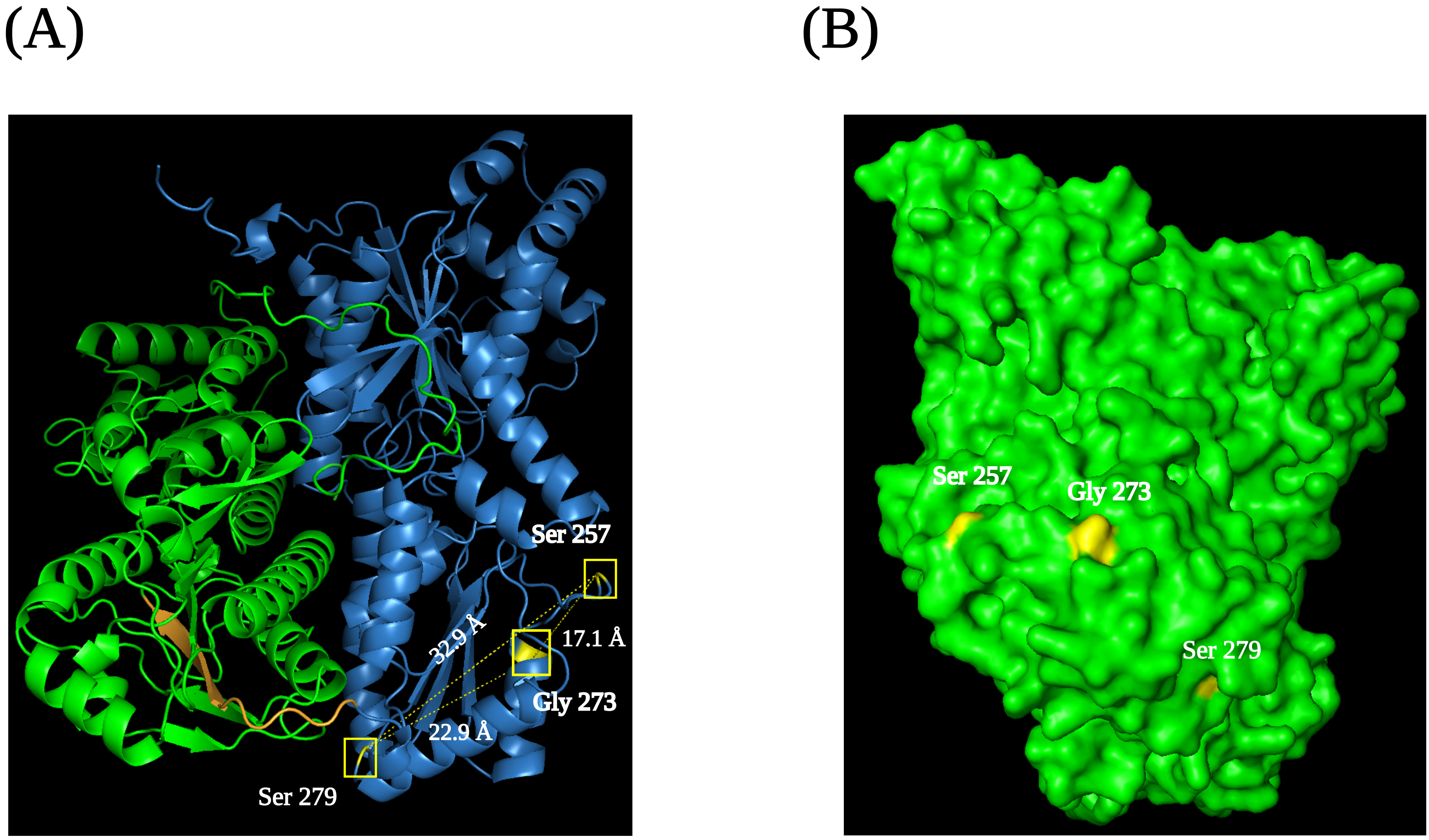

Figure S1. Spatial distances and surface distribution of positively selected sites in PFKP.
(A) Cartoon representation of PFKP showing its subunit organization (The N-terminal domain is shown in blue, the C-terminal domain is shown in green, and the linker region is highlighted in orange). Three positively selected sites (Ser257, Gly273, and Ser279) are indicated in yellow boxes, with yellow dashed lines denoting the spatial distances between them (32.9 Å for Ser257 – Ser279, 22.9 Å for Gly273 – Ser279, and 17.1 Å for Ser257 – Gly273).
(B) Molecular surface representation of PFKP (green). The three positively selected residues (Ser257, Gly273, and Ser279) are highlighted in yellow, demonstrating their exposure and locations within three distinct functional pockets on the protein surface.
