# Supplementary material for: Adaptive Evolution of the PFK Gene Family in Chinese Longsnout Catfish, Leiocassis longirostris
Source: Biology (Basel). 2026 Sep 1;15(17):1481. doi: 10.3390/biology15171481 (PMC13564638; doi:10.3390/biology15171481)
Supplement: Supplementary file 1 [file biology-15-01481-s001.zip › Table S1.pdf]

Table S1. Summary of genomic datasets, assembly versions, accession numbers, and dietary classifications for the seven representative fish species analyzed in this study

Species Name	Common Name	Database Source	Assembly Version	Accession Number	Date of Access
Ctenopharyngodon Idella	grass carp	NCBI	HZGC_01	GCF_019924925.1	Sep 14, 2021
Megalobrama amblycephala	blunt snout bream	NCBI	ASM1881202v1	GCF_018812025.1	Jun 9, 2021
Leiocassis longirostris	Chinese longsnout catfish	NCBI	previous study		[24]
Tachysurus fulvidraco	yellow catfish	NCBI	HZAU_PFXX_2.0	GCF_022655615.1	Mar 21, 2022
Danio rerio	zebrafish	NCBI	GRCz12tu	GCF_049306965.1	Apr 4, 2025
Acipenser ruthenus	sterlet	NCBI	fAciRut3.2	GCF_902713425.1	Feb 2, 2022
Callorhinchus milii	elephant shark	NCBI	IMCB_Cmil_1.0	GCF_018977255.1	Jun 21, 2021
